# Transcriptome sequencing and Mendelian randomization analysis identified biomarkers related to neutrophil extracellular traps in diabetic retinopathy

**DOI:** 10.3389/fimmu.2024.1408974

**Published:** 2024-10-17

**Authors:** Linlin Hao, Songhong Wang, Lian Zhang, Jie Huang, Yue Zhang, Xuejiao Qin

**Affiliations:** ^1^ Department of Ophthalmology, the Second Hospital of Shandong University, Cheeloo College of Medicine, Shandong University, Jinan, China; ^2^ Affiliated Eye Hospital of Shandong University of Traditional Chinese Medicine, Shandong University, Jinan, China; ^3^ Department of Operating Room, the Second Hospital of Shandong University, Cheeloo College of Medicine, Shandong University, Jinan, China

**Keywords:** neutrophil extracellular traps, transcriptome sequencing, mendelian randomization, pathway, diabetic retinopathy

## Abstract

**Summary:**

In the development of diabetic retinopathy (DR), neutrophil infiltration hastens the adhesion between neutrophils and endothelial cells, leading to inflammation. Meanwhile, neutrophil extracellular traps (NETs) produced by neutrophils could clear aging blood vessels, setting the stage for retinal vascular regeneration. To explore the mechanism of NETs-related genes in DR, the transcriptome of NETs from normal and DR individuals were analyzed with gene sequencing and mendelian randomization (MR) analysis. Five NETs-related genes were identified as key genes. Among these genes, CLIC3, GBP2, and P2RY12 were found to be risk factors for Proliferative DR(PDR), whereas HOXA1 and PSAP were protective factors. Further verification by qRT-PCR recognized GBP2, P2RY12 and PSAP as NETs-associated biomarkers in PDR.

**Purpose:**

To investigate neutrophil extracellular traps (NETs) related genes as biomarkers in the progression of diabetic retinopathy (DR).

**Methods:**

We collected whole blood samples from 10 individuals with DR and 10 normal controls (NCs) for transcriptome sequencing. Following quality control and preprocessing of the sequencing data, differential expression analysis was conducted to identify differentially expressed genes (DEGs) between the DR and NC groups. Candidate genes were then selected by intersecting these DEGs with key module genes identified through weighted gene co-expression network analysis. These candidate genes were subjected to mendelian randomization (MR) analysis, then least absolute shrinkage and selection operator analysis to pinpoint key genes. The diagnostic utility of these key genes was evaluated using receiver operating characteristic curve analysis, and their expression levels were examined. Additional analysis, including nomogram construction, gene set enrichment analysis, drug prediction and molecular docking, were performed to investigate the functions and molecular mechanisms of the key genes. Finally, the expression of key genes was verified by qRT-PCR and biomarkers were identified.

**Results:**

Intersection of 1,004 DEGs with 1,038 key module genes yielded 291 candidate genes. Five key genes were identified: HOXA1, GBP2, P2RY12, CLIC3 and PSAP. Among them, CLIC3, GBP2, and P2RY12 were identified as risk factors for DR, while HOXA1 and PSAP were protective. These key genes demonstrated strong diagnostic performance for DR. With the exception of P2RY12, all other key genes exhibited down-regulation in the DR group. Furthermore, the nomogram incorporating multiple key genes demonstrated superior predictive capacity for DR compared to a single key genes. The identified key genes are involved in oxidative phosphorylation and ribosome functions. Drug predictions targeting P2RY12 suggested prasugrel, ticagrelor, and ticlopidine as potential options owing to their high binding affinity with this key genes. The qRT-PCR results revealed that the results of GBP2, PSAP and P2RY12 exhibited consistent expression patterns with the dataset.

**Conclusion:**

This study identified GBP2, P2RY12 and PSAP as NETs-associated biomarkers in the development of PDR, offering new insights for clinical diagnosis and potential treatment strategies for DR.

## 1 Introduction

Diabetic retinopathy (DR) is the top microvascular complication of diabetes mellitus, affecting approximately 30% to 40% of diabetic patients (1, 2). Globally, DR afflicts over 100 million people, making it the leading cause of irreversible blindness in working-age adults (1, 3). The key characteristics of DR include capillary occlusion, retinal ischemia, and vascular leakage (4). Current treatment options for DR include intravitreal injection of anti-vascular endothelial growth factor (VEGF), retinal laser photocoagulation and vitrectomy surgery (5). Although these therapeutic interventions have been deemed safe and effective through clinical research, they only provide temporary control of DR progression. Moreover, retinal capillary damage is irreversible and abovementioned therapies carry the risk of elevated intraocular pressure and retinal ischemia (6–8). Therefore, it is a priority to explore and identify the underlying pathogenesis of DR, which may provide more effective disease management.

Under the stimulation of inflammatory mediators, neutrophils release chromatin, histones and neutrophil granule proteins, forming a network structure outside the cell that binds and kills pathogens. This network structure is called neutrophil extracellular traps (NETs) (9). Recent reports have shown elevated serum NETs levels in patients with proliferative diabetic retinopathy (PDR) (10, 11). Other evidence suggests that neutrophil infiltration accelerates the adhesion of neutrophil to endothelial cells and promotes inflammation. NETs produced by neutrophils play a role in removing aging-damaged blood vessels and promoting the regeneration of retinal vasculature (12). Neutrophil aggregation near neovascularization has been detected in retinal slices of human PDR patients, and typical NETs have been observed in vitreous and retinal tissues. These findings indicate that NETs participate in the process of DR (10, 13). However, it is unclear which NETs-related genes regulate the pathogenesis of DR.

To gain deeper insights into the potential role that NETs-related genes played in DR, we employed a statistical method known as Mendelian randomization (MR). Observational epidemiological studies are prone to reverse causation, confounding and various biases, which limits their ability to provide conclusive evidence (14, 15). MR analysis utilizes genetic variants as exposure indicators that are not influenced by conventional study designs (14). This method effectively eliminates the confounding effects of extraneous factors and enhances causal inference, contributing to a better understanding and prevention of adverse effects on human health caused by modifiable exposures (15, 16). Despite its proven utility, the application of MR in DR research remains limited. In this study, we utilized MR analysis to investigate the causal associations between NETs-related genes and DR.

To this end, we first identified differentially expressed genes (DEGs) between the DR and normal control (NC) groups through transcriptome sequencing and bioinformatics analysis. Subsequently, we examined and certified the associations between these identified markers and PDR. In our study, we revealed the significance of five NETs-related genes in the pathogenesis of PDR and provided novel strategies for DR control.

## 2 Materials and methods

This study was carried out in the Second Hospital of Shandong University and adhered to principles of the Declaration of Helsinki involving human subjects. The study was approved by the Ethics Committee of Scientific Research of the Second Hospital of Shandong University (Approval number: KYLL2024043). Signed consent was obtained from each participant.

### 2.1 Sample collection and RNA-sequencing analysis

Whole blood samples were collected from 10 patients with DR and 10 NC patients. The DR group consisted of patients who were diagnosed as PDR with vitreous hemorrhage whereas without retinal detachment or fibrous formation, with a mean age of 61.50 ± 7.13 years. It included 7 males and 3 females, all of whom had type 2 diabetes. All of the patients with diabetes were diagnosed according to the criteria of the American Diabetes Association (17). PDR was defined as the presence of neovascularization or fibrous proliferation of the disc or elsewhere on the retina (18). Patients with the following conditions were excluded: (1) severe systemic diseases such as metabolic syndrome (excluding type 1 diabetes), ongoing infection or autoimmune diseases and malignant tumors; and (2) any other ocular disease, such as glaucoma, high myopia, retinal diseases, or a history of previous ocular surgery (except mild cataract) (19).

The NC group contained patients who underwent routine cataract surgery at the hospital. The patients had a mean age of 59.20 ± 10.83 years and included 6 males and 4 females. The patients would be excluded if they were found to suffer from ocular diseases other than age-related cataract, as well as participants with diabetes, hyperthyroidism, hypertension, immune-related diseases, and cancer. The specific sample information can be found in **Supplementary Table S1**.

The manufacturer’s protocol was followed to extract total RNA using TRIzol (Invitrogen, CA, USA). The quantity and quality of total RNA were assessed using NanoDrop ND-1000 (NanoDrop, Wilmington, DE, USA) to ensure its integrity. Subsequently, the assessment of RNA integrity was conducted utilizing a Bioanalyzer 2100 (Agilent, CA, USA) and verified through agarose electrophoresis. The samples were considered acceptable if they met the following criteria: concentration > 50 ng/μL, RNA integrity number (RIN) > 7.0, optical density (OD) 260/280 > 1.8, and total RNA > 1μg. Based on the guidelines provided by the manufacturer, the library was prepared for Illumina sequencing using Hieff NGS Ultima Dual-mode mRNA Library Prep Kit. Importantly, these libraries were sequenced on the Illumina Novaseq 6000 platform in a pair-end (PE) 150 mode. The overview of this study design was shown in **Supplementary Figure S1**.

### 2.3 Preprocessing of sequencing data

The quality of the sequencing data was evaluated using FastQC software. The original data were preprocessed using Trimmomatic to filter out low-quality data and remove pollution and joint sequences, resulting in clean data. The filtered clean sequencing data were then compared to the reference genome using the hisat2 comparison tool with default parameters. After the analysis, the gene expression matrix was obtained for subsequent analysis.

### 2.4 Identification and enrichment analysis of DEGs

The DESeq2 package (v 1.36.0) (20) was utilized to compare gene expression levels between the DR and NC groups. DEGs were identified with screening conditions of p < 0.05 and |log2FoldChange(FC)| > 0.5. Volcano plots were generated using ggplot2 (v 3.4.0) (21) to visualize DEGs, heatmaps were generated using ComplexHeatmap (v 2.12.1) (22) to visualize the expression patterns of the top 10 up- and downregulated DEGs. Enrichment analyses based on the Gene Ontology (GO) and Kyoto Encyclopedia of Genes and Genomes (KEGG) databases were performed using the clusterProfiler package (v 4.4.4) (23) and the org.Hs.eg.db (v 3.15.0) (24) human gene annotation package (*p* adj < 0.05). The GO system comprised three components: biological process (BP), molecular function (MF) and cellular component (CC). The treemap package (v 2.4-3) was used to generate a tree diagram illustrating the enrichment results.

### 2.5 Screening of NETs related module genes

A total of 257 NETs-related genes were obtained from published literature (25, 26). NETs scores were calculated for each sample using the GSVA package (v 1.38.2) (27), the differences in NETs scores between DR and NC groups were compared using the Wilcoxon test. The results were visualized using gghalves (v 0.1.4) to plot cloud and rain maps. Based on all gene expression data, NETs scores were used as trait data, a weighted gene co-expression network analysis (WGCNA) was performed using WGCNA package (v 1.72-1) (28) to screen for the modules and their genes that were most relevant to NETs scores.

Cluster analysis was implemented on the samples to determine the necessity of removing outlier samples for accurate subsequent analyses. The optimal soft threshold (β) was determined to maximize the adherence of gene interactions to the scale-free distribution. According to the standard of the hybrid dynamic tree cutting algorithm, a threshold of 200 genes per gene module was established in order to facilitate clustering of genes into distinct modules. The correlation between each module and the NET score was analyzed with the criteria of |cor| > 0.3 and *p* < 0.05 to identify gene modules associated with the NET score trait. The module exhibiting the highest relevance to the NET scores was defined as the key module and included key module genes.

### 2.6 The identification and functional investigation of candidate genes

The ggVennDiagram package (v 1.2.2) (29) was utilized to intersect DEGs and key module genes to identify differentially expressed NETs-related genes, which were recorded as candidate genes. To further investigate the functions of the candidate genes, functional enrichment analysis was implemented via the Metascape database (https://metascape.org/gp/index.html#/main/step1). On the other hand, a protein-protein interaction (PPI) network was constructed based on the STRING (https://string-db.org) website so as to explore whether there were interactions between candidate genes (confidence = 0.4). The network was visualized by Cytoscape software (v 3.9.1) (30).

### 2.7 Screening of instrumental variables in MR

MR studies need to comply with three basic principles: (1) there is a durable and significant correlation between IVs and exposure factors; (2) IVs are not associated with confounding factors; (3) IVs can only influence outcome variables through exposure factors. The DR-related Genome-wide association study (GWAS) dataset (trait ID: finn-b-DM RETINOPATHY EXMORE) was provided by Integrative Epidemiology Unit (IEU) Open GWAS (https://gwas.mrcieu.ac.uk/) database. This dataset included 190,594 European samples (cases: controls = 14,584: 176,010) and 16,380,347 single nucleotide polymorphisms (SNPs).

In MR analysis, each candidate gene was treated as an exposure factor, DR was treated as the result. The exposure factors were examined and IVs were filtered using the extract instruments function in the TwoSampleMR package (version 0.5.6) (31). SNPs significantly related to exposure factors (*p* < 5×10^-8^) were searched, while SNPs showing linkage disequilibrium (LD) (clump=TRUE, r^2^ = 0.001, kb=10000) were excluded. The directionality test function in the TwoSampleMR package was employed to test the directionality of the exposure factors. Furthermore, the F statistic was calculated to evaluate the strength of each IV. If the F statistic was greater than 10, it was considered that there was no weak instrumental bias, indicating a strong predictive potential of the IV for the outcome.

### 2.8 MR analysis between candidate genes and DR

The SNPs of exposure factors and outcomes were unified by the function harmonise data. MR analysis was then performed using the mr function combined with five algorithms: MR Egger (32), weighted median (33), inverse variance weighting (IVW) (34), simple mode (35) and weighted mode (36). The IVW method was deemed the most crucial among the five methods and its outcomes were conclusive. A *p* value less than 0.05 indicated a causal relationship between candidate genes and DR. A b value greater than 0 represented a risk factor, while a b value less than 0 indicated a protective factor. The MR results were visualized using scatter plots, funnel plots and forest plots.

Sensitivity analysis was conducted to assess the reliability of the MR results. Initially, the Cochran’s Q test was employed to evaluate the presence of heterogeneity in the tests, with a *p* value greater than 0.05 indicating no significant heterogeneity. Subsequently, a horizontal pleiotropy test was conducted to determine the presence of confounding factors (*p* > 0.05). Eventually, we employed a Leave-One-Out (LOO) analysis to systematically remove each SNP and assess whether the IVW method was affected by any particular individual SNPs. The genes with potential causal relationship with DR in MR analysis were defined as candidate key genes for subsequent analysis.

### 2.9 Access to key genes

The candidate key genes identified from MR analysis were included in the least absolute shrinkage and selection operator (LASSO) regression analysis for further screening. Specially, the LASSO regression analysis was implemented via glmnet package (v 4.1-6) (37). The genes identified as key genes had the lowest cross-validation error rate. Additionally, the diagnostic ability of each key genes was assessed by plotting the receiver operating characteristic (ROC) curve using pROC package (v 1.17.0.1) (38), larger values of area under the curve (AUC) representing more accurate diagnostic ability of the key genes for DR. Furthermore, the expression of key genes was assessed between DR and NC groups, the results were visualized using ggpubr (v 0.4.0) (39) to generate a box plot. Spearman correlation analysis was performed among the key genes and the results were demonstrated using circle plots created with the circlize package (v 0.4.15) (40).

### 2.10 Construction and evaluation of nomogram

The key genes obtained above were employed to construct a nomogram through rms package (v 6.2-0) (41) to facilitate the clinical judgment of the risk rate of DR. The nomogram assigned a score to each key genes, with each score corresponding to a specific key genes. Subsequently, the risk rate of DR was predicted based on the cumulative score. A higher score indicated an increased risk rate. In addition, ROC curves, decision curves and clinical impact curves were plotted to evaluate the predictive value of nomogram. Specially, the decision curve analysis (DCA) was generated via rmda package (v 1.6).

### 2.11 Gene set enrichment analysis

To further explore the pathways and functions associated with the key genes, GSEA was implemented based on the KEGG database via clusterProfiler package (v 4.4.4). First, the correlation coefficients between the key genes and the remaining genes in the dataset were determined. The genes were then sorted based on these correlation coefficients. Subsequently, GSEA was conducted according to the sorting results, with a significance threshold of *p* adj < 0.05). Additionally, the GSEA ridge plot was drawn using GseaVis package (v 0.0.5) to visualize the enrichment results.

### 2.12 Drug prediction and molecular docking

The Drug-Gene Interaction Database (DGIdb) (https://dgidb.genome.wustl.edu/) was utilized to predict key genes-related drugs with potential therapeutic effects in DR. Drugs reported in the literature were selected for molecular docking analysis. The 3D Conformer structures of the drugs was retrieved from PubChem database (https://pubchem.ncbi.nlm.nih.gov/). Meanwhile, the crystal structure of the key genes with Protein Data Bank (PDB) ID 4ntj was obtained from the PDB database (https://www1.rcsb.org/). Subsequently, molecular docking was performed using AutoDock vina and visualized by PyMol software (v 2.5).

### 2.13 Quantitative real-time polymerase chain reaction

To further confirm the results of the public database analysis, we collected five paired DR and NC whole blood samples and implemented RNA isolation and quantitative real-time polymerase chain reaction (qRT-PCR). Total RNA of 10 samples was separated by the TRIzol (Ambion, Austin, USA) based on the manufacturer’s guidance. The inverse transcription of total RNA into cDNA was implemented by using the SureScript-First-strand-cDNA-synthesis-kit (Servicebio, Wuhan, China) based on the producer’s indication. Then, qPCR was carried out utilizing the 2xUniversal Blue SYBR Green qPCR Master Mix (Servicebio, Wuhan, China) under the direction of the manual. The primer sequences for PCR were tabulated in **Table 1**. The expression was uniformized to the internal reference GAPDH and computed employing the 2^−ΔΔCt^ method. Finally, key genes with qRT-PCR results consistent with the dataset were identified as biomarkers.

**Table 1:** Primer information.

Primer	Sequence(5’-3’)	
HOXA1 F	GGAAGCAGACCCACCAAGAA	
HOXA1 R	TCACTTGGGTCTCGTTGAGC	
GBP2 F	CTTCAGGAACAGGAACGCCT
GBP2 R	GTTTCTTGGGGAGAGGGAGC
P2RY12 F	CACTGCTCTACACTGTCCTGT
P2RY12 R	AGTGGTCCTGTTCCCAGTTTG
CLIC3 F	GCTGTTTGTCAAGGCGAGTG
CLIC3 R	CCAGCGTCTCCTCCAGAAAG
PSAP F	GAAATCCCTTCCCTGCGACA
PSAP R	AGGTTGAGAGCAGAGCACAC
GAPDH F①	CGAAGGTGGAGTCAACGGATTT
GAPDH R①	ATGGGTGGAATCATATTGGAAC
GAPDH F②	CGAAGGTGGAGTCAACGGATTT
GAPDH R②	ATGGGTGGAATCATATTGGAAC

### 2.14 Statistical analysis

The data were processed and analyzed using R software (version 4.2.1). The nonparametric Wilcoxon test was employed to assess differences between different groups, with a *p* value less than 0.05 considered to statistical significance.

## 3 Results

### 3.1 A total of 1,004 DEGs were associated with inflammation-related signaling pathways in DR

**Figure 1:**
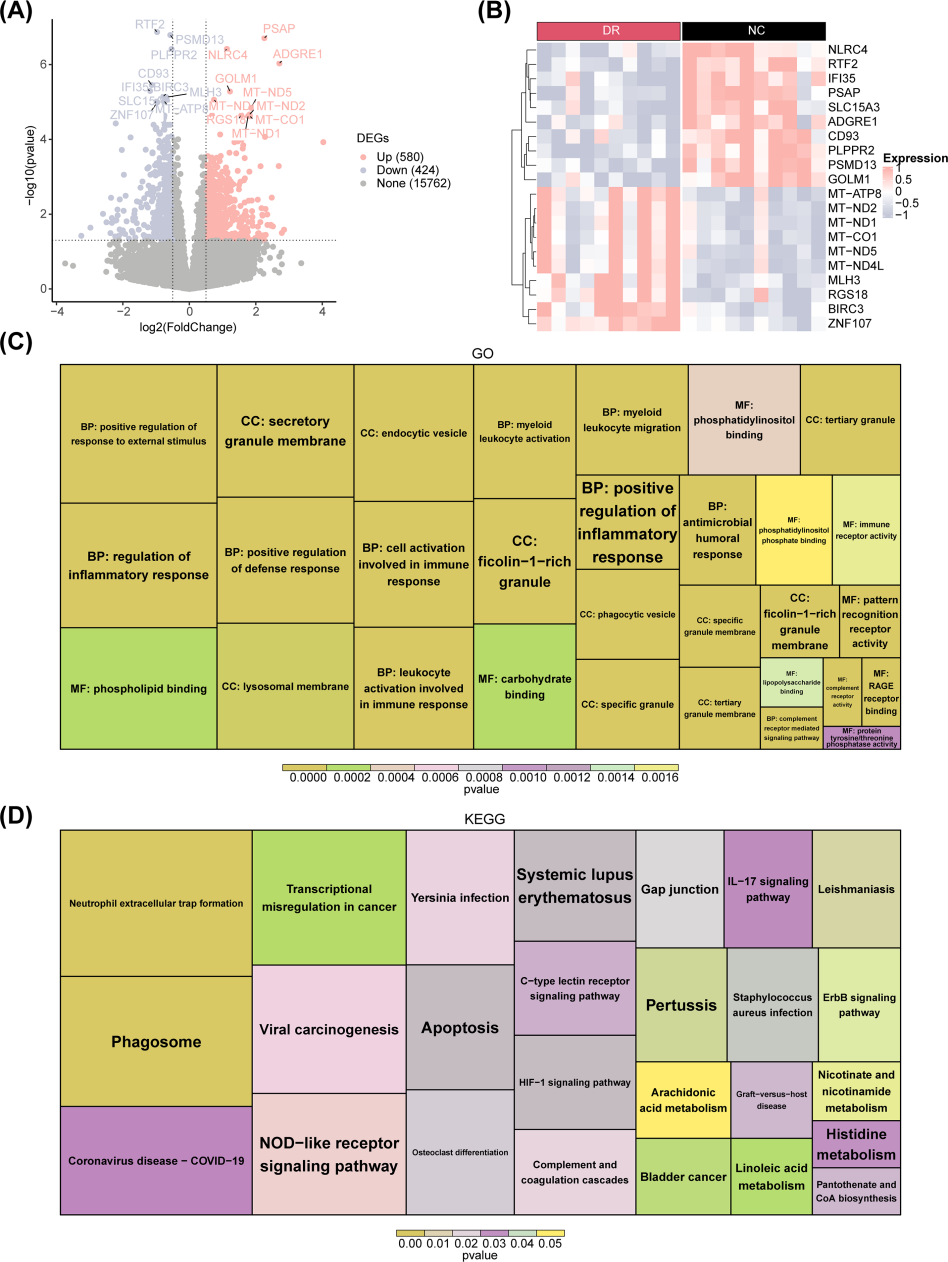
Differential expression analysis between the DR and NC groups. **(A)** Volcano plot displaying differentially expressed genes (DEGs) between DR patients and healthy controls for combined expression profiling. Pink nodes indicate upregulated DEGs; blue nodes indicate downregulated DEGs; gray nodes indicate genes that are not significantly expressed. **(B)** Heatmap plot of the top 10 DEGs. Red indicates DR samples, black indicates normal control samples, pink indicates high gene expression, and blue indicates low gene expression. **(C)** GO enrichment plot. **(D)** KEGG enrichment plot.

Differential expression analysis generated 1,004 DEGs between DR and NC groups. Among these DEGs, 580 were up-regulated in DR samples, while 424 were downregulated, in addition, heatmaps were drawn to visualise the expression patterns of up- and down-regulated TOP10 differential genes. (**Figures 1A, B**). Subsequently, enrichment analysis was conducted to probe the signaling pathways involved in these DEGs. The results revealed that these DEGs were enriched in 741 GO entries (including 590 BPs, 72 CCs and 79MFs) and 26 KEGG pathways. Specially, these enriched GO entries included cell activation involved in immune response, regulation of inflammatory response, leukocyte activation involved in immune response, etc (**Figure 1C**). Meanwhile, KEGG enrichment results showed that these DEGs were enriched mainly in NOD-like receptor signaling pathway, IL-17 signaling pathway and others (**Figure 1D**).

### 3.2 The identification of 1,038 key module genes was accomplished

**Figure 2:**
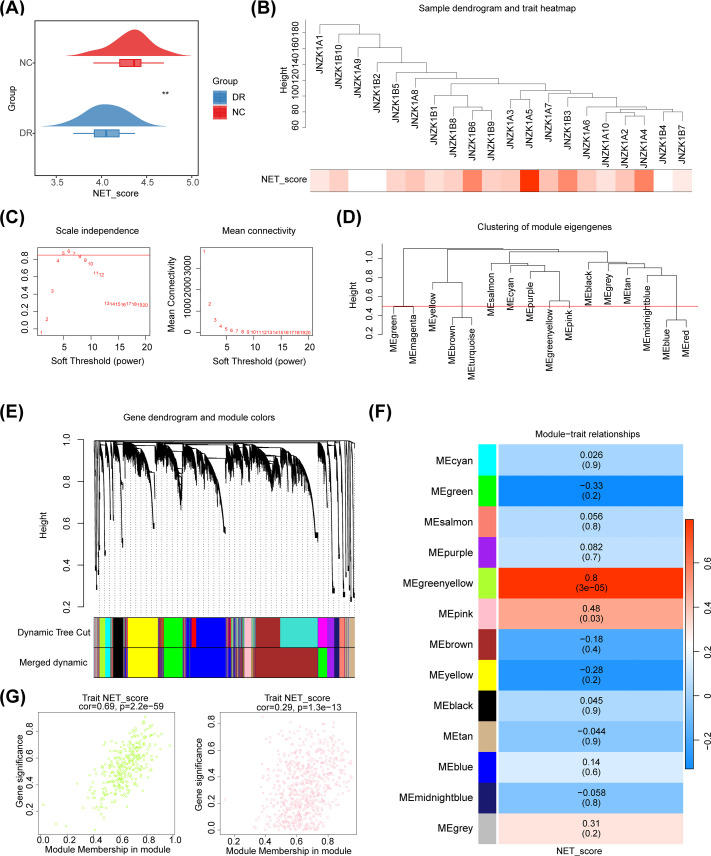
Screening of NETs related module genes. **(A)** Cloud and rain map. Red indicates control samples, and blue indicates normal DR samples. The NETs were significantly lower in the DR group. **(B)** Sample dendrogram and trait heatmap plot. Cluster analysis revealed that there were no outliers in the samples. **(C)** Selection of the best soft threshold. **(D)** Clustering tree based on the module eigengenes of the modules. **(E)** Hierarchical cluster dendrogram of identified genes. Each color represents a module, and the vertical line represents a gene. **(F)** Heatmap of the correlations between module traits and the NET score. Each color represents a module, and the box contains the correlation coefficient and p value (p value in parentheses). Blue represents a negative correlation, and red represents a positive correlation. **(G)** The scatter plot of the correlation between key module genes and NETs score traits.

The results of the GSVA demonstrated a significant difference in NETs score between DR and NC groups. The NETs score was significantly lower in the DR group than in the NC group, indicating that NETs have an impact on the occurrence and development of DR (**Figure 2A**). Therefore, the NETs score could be used as a trait to find the key module genes related to it through WGCNA. Cluster analysis revealed that there were no outliers in the samples (**Figure 2B**). When the ordinate scale-free R^2^ crossed the threshold of 0.85 (red line), the first soft threshold β was determined to be 5, and the mean connectivity also tended to 0, indicating that the network approximated the scale-free distribution at this time. Therefore, the best soft threshold β was selected as 5 (**Figure 2C**). Furthermore, 16 modules were obtained (**Figure 2D**). After merging with a similarity of 0.5, 13 modules were ultimately obtained (**Figure 2E**). The results of correlation analysis demonstrated that MEgreenyellow (cor = 0.8, p = 3× 10^-5^) and MEpink (cor = 0.48, p = 0.03) modules were strongly correlated with NETs scores (**Figure 2F**). Therefore, these two modules were defined as key modules, containing 1,038 key module genes. The scatter plot of the correlation between key module genes and NETs score traits was shown in **Figure 2G**.

### 3.3 The 291 candidate genes were mainly involved in inflammation and immune-related signaling pathways in DR

**Figure 3:**
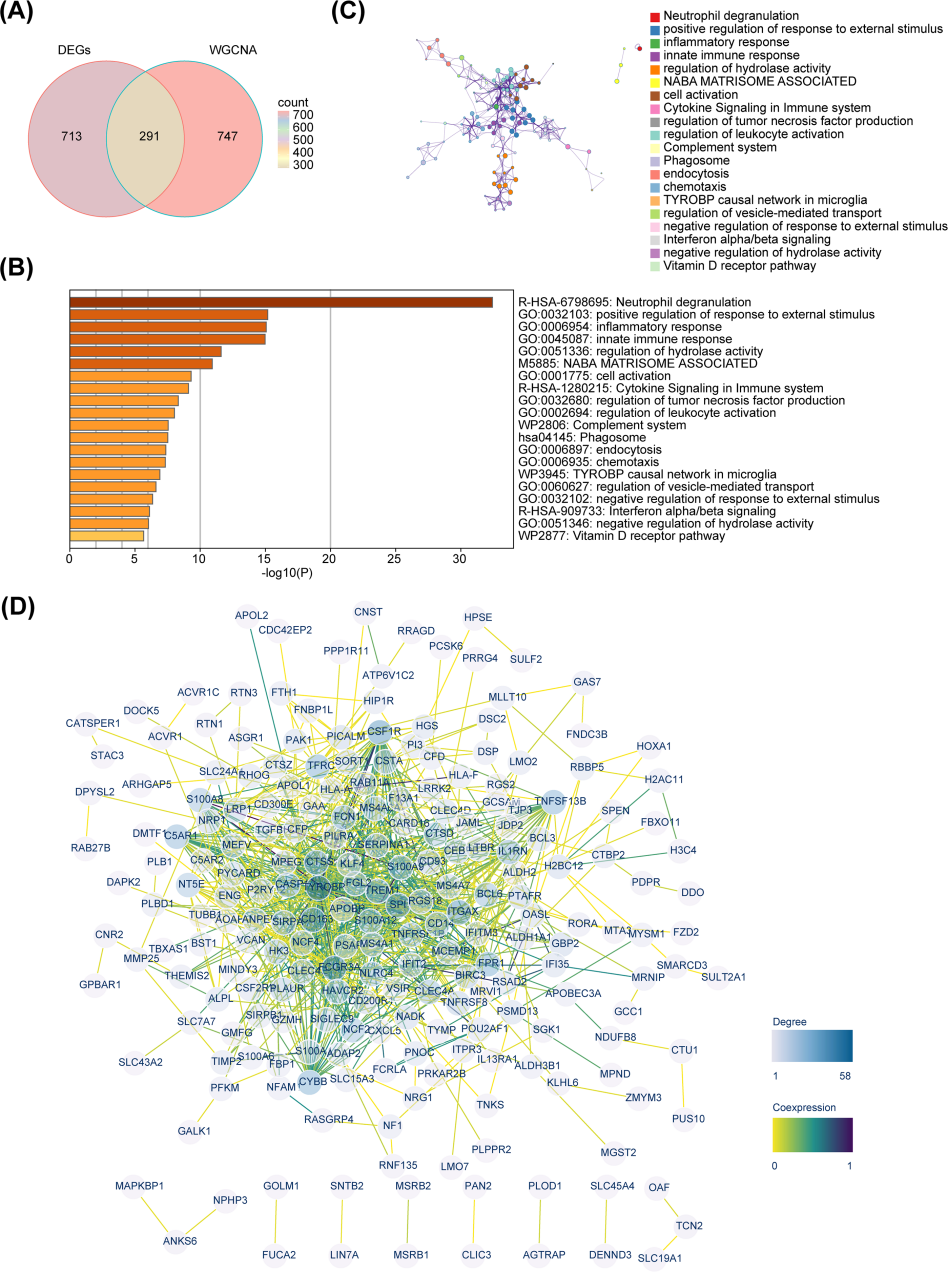
Identification and functional investigation of candidate genes. **(A)** Venn diagram. **(B)** Enrichment bar chart of 291 candidate genes. **(C)** Metascape enrichment interaction network. **(D)** Protein-protein interaction network.

By overlapping 1,004 DEGs and 1,038 key module genes, 291 candidate genes were identified (**Figure 3A**). Enrichment analysis revealed that these candidate genes were mainly enriched in inflammatory and immune-related signaling pathways, such as inflammatory response, innate immune response, cytokine signaling in immune system, and regulation of leukocyte activation (**Figure 3B**). Moreover, we explored the links between these pathways and found that each function may have the same genes and functions interact with each other (**Figure 3C**). Based on a confidence level of 0.4, a PPI network consisting of 222 nodes and 820 edges was constructed. Notably, C5AR1, YROBP, FCGR3A, CYBB and other candidate genes exhibited interactions with multiple genes (**Figure 3D**).

### 3.4 The nomogram based on key genes exhibited superior diagnostic efficacy for DR

**Figure 4:**
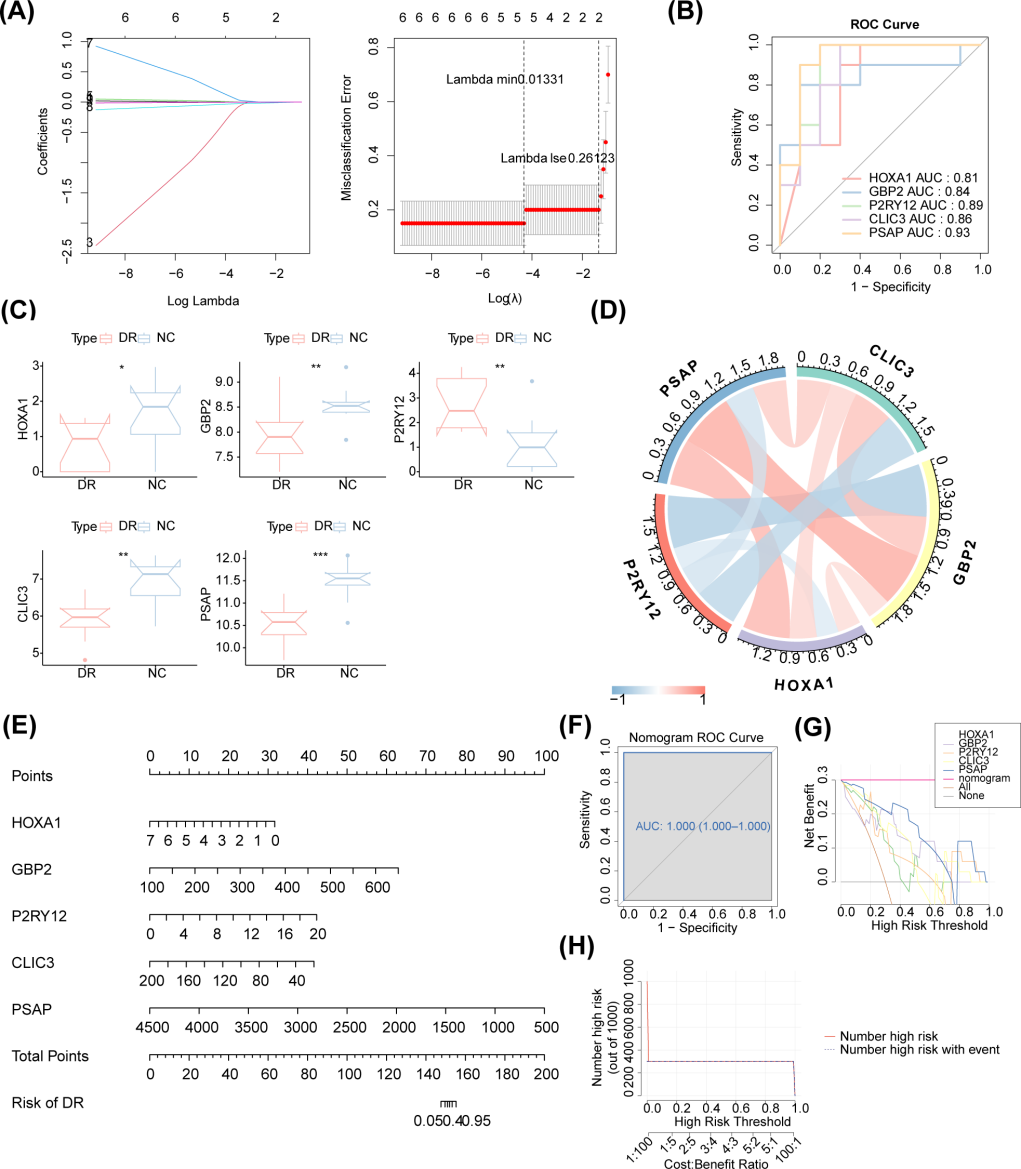
Access to biomarkers. **(A)** LASSO regression analysis. The horizontal axis deviation represents the proportion of residuals explained by the model, showing the relationship between the number of feature genes and the proportion of residuals explained (dev). The vertical axis represents the coefficient of genes (left); the horizontal axis represents log (Lambda), and the vertical axis represents the error of cross-validation (right). In practical analysis, we hope to find the position with the smallest cross-validation error. In the right graph, the dotted line on the left is the position with the smallest cross-validation error. Based on this position (lambda. min), the corresponding horizontal axis log (Lambda) is determined. The number of feature genes is displayed above, and the optimal log (Lambda) value is found. The corresponding gene and its coefficient are shown in the left graph, as well as the proportion of residuals explained by the model. **(B)** The ROC curve of these five biomarkers. The AUC values of these five biomarkers exceeded 0.7. **(C)** Boxplot of the expression of these five biomarkers. P2RY12 exhibited upregulation, and all other biomarkers exhibited downregulation in the DR group. **(D)** Chord diagram of the five identified biomarkers. There was a highly significant negative correlation between GBP2 and P2RY12 (cor = -0.641, p = 0.002), while a significant positive correlation was observed between GBP2 and PSAP (cor = 0.660, p = 0.002). **(E)** The nomogram of the five identified biomarkers. **(F)** The nomogram ROC curve. **(G)** Decision curve analysis. **(H)** Clinical impact curve analysis.

The MR analysis results demonstrated a causal relationship between 11 exposure factors and DR (IVW *p* < 0.05) (**Table 2**). Among them, CLIC3 (b = 0.327, *p* = 5.32E-05), GBP2 (b = 0.303, *p* = 0.013), HLA-A (b = 0.250, *p* = 0.004), SPI1 (b = 0.109, *p* = 0.001), P2RY12 (b = 0.085, *p* = 0.010) and OASL (b = 0.054, *p* = 0.005) were identified as risk factors for DR, whereas DENND3 (b = -0.065, *p* = 0.045), HLA-F (b = -0.150, *p* = 0.015), SLC22A15 (b = -0.156, *p* = 0.022), HOXA1 (b = -0.172, *p* = 0.012) and PSAP (b = -0.189, *p* = 1.35E-07) were found to be protective factors for DR (b < 0, *p* < 0.05). **Supplementary Table S2** provides the F statistics for these 11 candidate key genes. These 11 candidate key genes were subsequently subjected to LASSO analysis for screening purposes. When lambda_min_ was 0.01331, five genes were screened by LASSO regression analysis, which were HOXA1, GBP2, P2RY12, CLIC3 and PSAP (**Figure 4A**). These genes were defined as key genes for subsequent analysis. The ROC curve demonstrated that the AUC values of these five key genes exceeded 0.7, indicating their robust diagnostic potential for DR (**Figure 4B**). Furthermore, a comparison of the expression levels of these five key genes between the DR and NC groups revealed a significant disparity. With the exception of P2RY12, all other key genes exhibited significantly downregulated expression in the DR group (**Figure 4C**). The correlation analysis indicated a highly significant negative correlation between GBP2 and P2RY12 (cor = -0.641, *p* = 0.002), while a significant positive correlation was observed between GBP2 and PSAP (cor = 0.660, *p* = 0.002) (**Figure 4D**; **Supplementary Table S3**).

**Table 2:** MR analysis between 11 exposure factors and DR.

gene	id.exposure	nsnp	b	*p*val	log2FoldChange	*p* value
CLIC3	eqtl-a-ENSG00000169583	4	0.326987	0	-1.010601402	0.0000532
GBP2	eqtl-a-ENSG00000162645	5	0.303424	0	-0.532966618	0.013069697
HLA-A	eqtl-a-ENSG00000206503	7	0.250395	0	-2.356635528	0.003645471
SPI1	eqtl-a-ENSG00000066336	5	0.10926	0	-0.711153491	0.000898097
P2RY12	eqtl-a-ENSG00000169313	9	0.085127	0.018	1.664665635	0.010063468
OASL	eqtl-a-ENSG00000135114	9	0.054271	0.033	-0.629893107	0.004634299
DENND3	eqtl-a-ENSG00000105339	5	-0.064965	0.036	-0.571122127	0.044903216
HLA-F	eqtl-a-ENSG00000204642	8	-0.149819	0	-2.196836641	0.014580304
SLC22A15	eqtl-a-ENSG00000163393	3	-0.156494	0.008	-0.915905355	0.021889917
HOXA1	eqtl-a-ENSG00000105991	4	-0.172059	0	-1.634093135	0.012697427
PSAP	eqtl-a-ENSG00000197746	3	-0.189472	0.002	-0.970284516	0.000000135

Based on these five identified key genes, a nomogram was constructed (**Figure 4E**). The AUC value of the ROC curve was 1, indicating that the nomogram outperformed gene prediction alone (**Figure 4F**). Meanwhile, the decision curve analysis demonstrated that the nomogram model exhibited significant benefits within the high-risk threshold range of 0-1, surpassing the clinical utility of the HOXA1, GBP2, P2RY12, CLIC3 and PSAP curves (**Figure 4G**). Furthermore, the clinical impact curve further substantiated the superior predictive capability of the nomogram model (**Figure 4H**).

### 3.5 Notable causal relationship between key genes and DR

**Figure 5:**
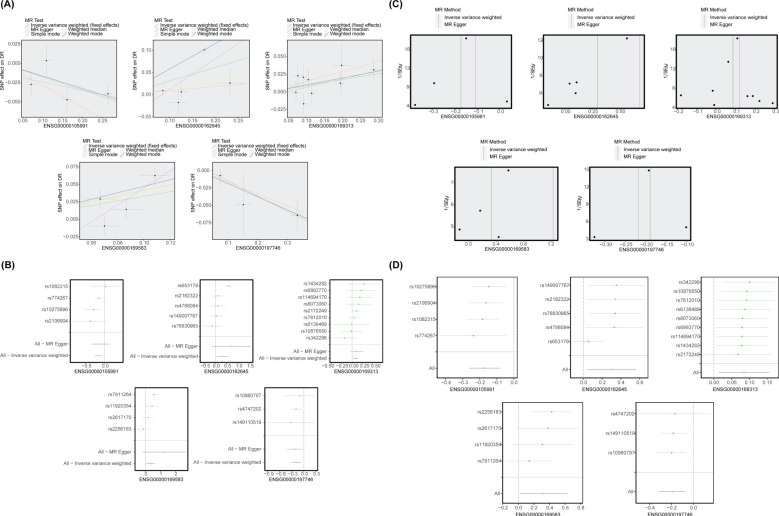
MR analysis between biomarkers and DR. **(A)** The scatter plot of the MR analysis. CLIC3, GBP2, and P2RY12 had positive slopes, whereas HOXA1 and PSAP had negative slopes. **(B)** The forest plot of the MR analysis. CLIC3, GBP2, and P2RY12 increased the risk of DR, whereas HOXA1 and PSAP decreased the risk of DR. **(C)** The funnel plot of the MR analysis. **(D)** LOO analysis. There was no significant alteration in the results upon exclusion of each SNP.

MR analysis revealed that CLIC3 (b = 0.327, *p* = 5.32E-05), GBP2 (b = 0.303, *p* = 0.013), and P2RY12 (b = 0.085, *p* = 0.010) were risk factors associated with DR, while HOXA1 (b = -0.172, *p* = 0.012) and PSAP (b = -0.189, *p* = 1.35E-07) exhibited protective effects against the development of DR. The scatter plot showed that CLIC3, GBP2 and P2RY12 had positive slopes, indicating an increased risk for DR. Conversely, HOXA1 and PSAP exhibited a negative slope indicating their potential protective role against DR (**Figure 5A**). The forest plot demonstrated that CLIC3, GBP2 and P2RY12 increased the risk of DR, whereas HOXA1 and PSAP decreased the risk of DR (**Figure 5B**). The funnel plot further reflected that the MR analysis conformed to Mendel ‘s second law (**Figure 5C**). Sensitivity analysis was employed to assess the robustness of the MR results. The heterogeneity test revealed that CLIC3 and GBP2 exhibited significant heterogeneity with DR (the *p*-value of Cochran’s Q test in IVW results was less than 0.05). However, it is important to note that this observed heterogeneity did not impact the established causal relationships (**Table 3**). The *p*-values of the five key genes all exceeded 0.05, indicating the absence of horizontal pleiotropy between key genes and DR (**Table 4**). Furthermore, the LOO analysis showed no significant alteration in the results upon exclusion of each SNP, suggesting the absence of any substantial bias point (**Figure 5D**). In summary, these findings implied a substantial causal association between key genes and DR.

**Table 3:** Heterogeneity test of the five identified biomarkers.

id.exposure	method	Q	Q_df	Q_*p*val
eqtl-a-ENSG00000169583	MR Egger	5.425	2	0.066
eqtl-a-ENSG00000169583	IVW	9.721	3	0.021
eqtl-a-ENSG00000162645	MR Egger	16.941	3	0.001
eqtl-a-ENSG00000162645	IVW	21.818	4	0.000
eqtl-a-ENSG00000169313	MR Egger	6.086	7	0.530
eqtl-a-ENSG00000169313	IVW	6.318	8	0.612
eqtl-a-ENSG00000105991	MR Egger	2.618	2	0.270
eqtl-a-ENSG00000105991	IVW	3.047	3	0.384
eqtl-a-ENSG00000197746	MR Egger	0.278	1	0.598
eqtl-a-ENSG00000197746	IVW	0.407	2	0.816

**Table 4:** Horizontal pleiotropy test of the five identified biomarkers.

id.exposure	egger_intercept	se	*p*val
eqtl-a-ENSG00000169583	-0.078	0.062	0.335
eqtl-a-ENSG00000162645	-0.058	0.062	0.421
eqtl-a-ENSG00000169313	-0.006	0.012	0.644
eqtl-a-ENSG00000105991	-0.013	0.024	0.625
eqtl-a-ENSG00000197746	0.007	0.019	0.781

### 3.6 Oxidative phosphorylation and ribosome were functional pathways related to the identified key genes

**Figure 6:**
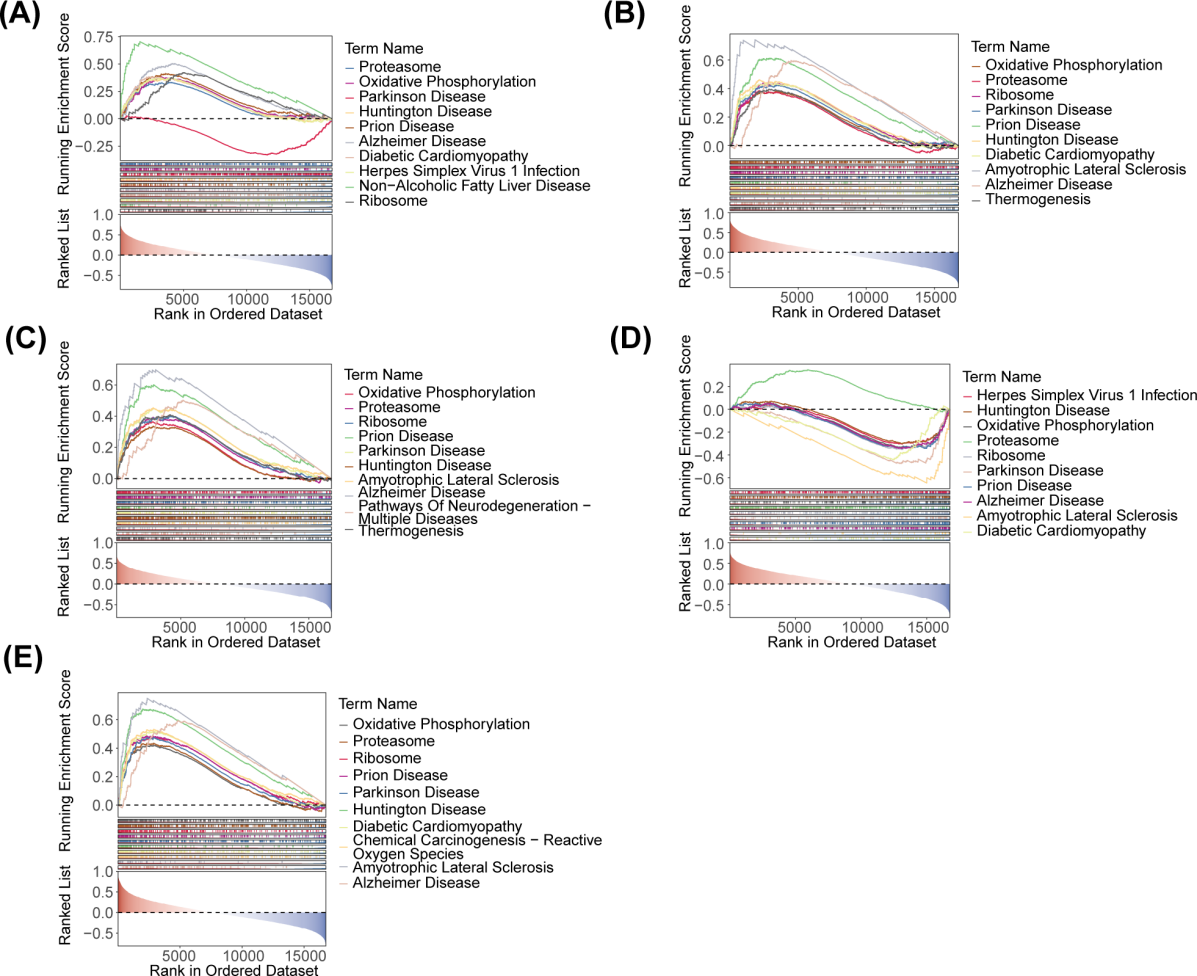
GSEA ridge plot of five identified biomarkers. **(A)** HOXA1. **(B)** GBP2. **(C)** P2RY12. **(D)** CLIC3. **(E)** PSAP.

GSEA was conducted to further explore the pathways associated with the key genes and their functions. The five key genes were associated with oxidative phosphorylation and ribosome functioning pathways. These pathways were also implicated in Parkinson Disease, Diabetic Cardiomyopathy, Herpes Simplex Virus 1 Infection, etc. (**Figures 6A-E**).

### 3.7 Identification of biomarkers by qRT-PCR

**Figure 7:**
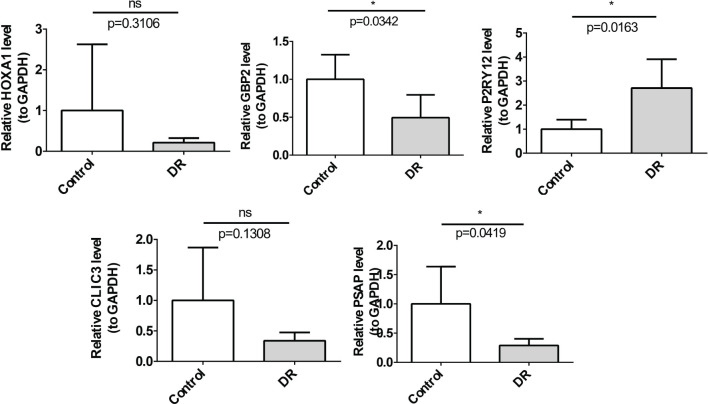
The relative mRNA expression of 5 biomarkers in the DR and control groups determined by qRT-PCR. The mRNA levels of GBP2 (*p*=0.0342) and PSAP (*p*=0.0419) were down-regulated, P2RY12 (*p*=0.0163) was up-regulated *p<0.05.

The qRT-PCR results revealed that among the five key genes, GBP2, PSAP and P2RY12 whose expression results were consistent with the expression pattern of the dataset were identified as biomarkers. Specifically, GBP2 and PSAP were significantly downregulated in the DR group, while P2RY12 was significantly upregulated. The expression trends of HOXA1 and CLIC3 aligned with the dataset, however, no significant difference was observed between the DR and NC groups (**Figure 7**).

### 3.8 Stable binding between biomarkers and drugs

**Figure 8:**
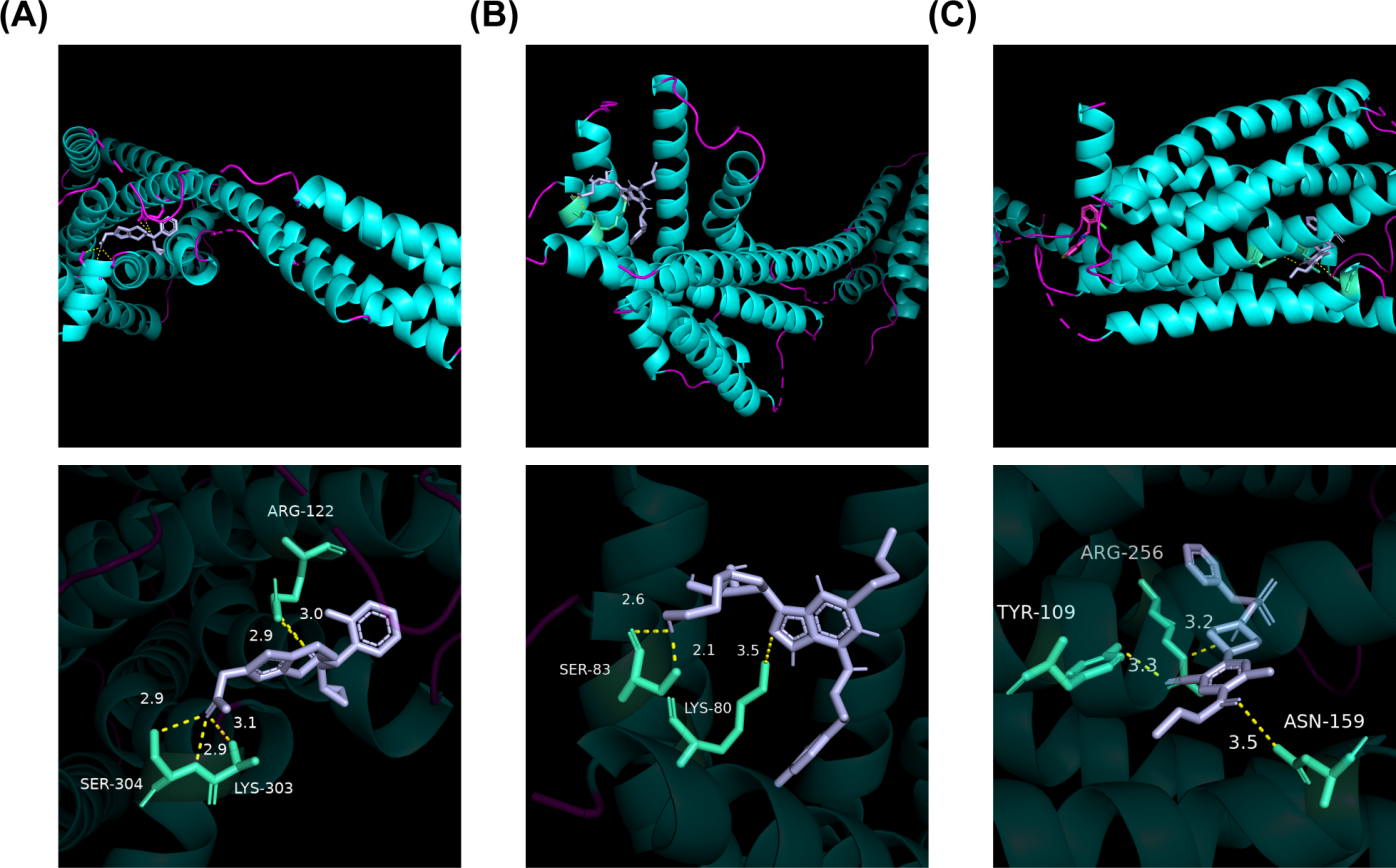
Molecular docking plot of the three drugs with P2RY12. **(A)** Molecular docking plot of 4 ntj with PRASUGREL. ARG-122, LYS-303, and SER-304 exhibited hydrogen bond interactions with PRASUGREL molecules. The docking affinity between this active molecule and P2RY12 was -6.8 kcal/mol. **(B)** The molecular docking plot of 4 ntj with TICAGRELOR. SER-83 and LYS-80 engaged in hydrogen bonding interactions with TICAGRELOR molecules. The docking affinity between this active molecule and P2RY12 was measured to be -8.2 kcal/mol. **(C)** The molecular docking plot of 4 ntj with TICLOPIDINE. TYR-109, ASN-159, ARG-256, and other residues exhibited hydrogen bond interactions with the TICLOPIDINE molecule. The docking affinity between this active molecule and P2RY12 was -6.6 kcal/mol.

Only the drugs corresponding to P2RY12 were retrieved from DGIdb. Among them, prasugrel (42, 43), ticagrelor (44) and ticlopidine (42, 45) had associated references. Consequently, molecular docking of these three drugs with P2RY12 was conducted (**Figure 8**). These results indicated that there was a robust binding ability between biomarkers and predicted drugs, which might provide potential drug targets for the treatment of DR.

## 4 Discussion

In the initial phase of this investigation, we identified the five most interesting NETs-related genes through MR and LASSO analysis. The diagnostic performance of these five NETs-related genes was excellent for PDR, as demonstrated by ROC curve analysis. CLIC3, GBP2 and P2RY12 were identified as risk factors for PDR, while HOXA1 and PSAP were found to have protective functions. P2RY12 was upregulated in the DR group, while the other key genes were downregulated. Three of key genes, GBP2, P2RY12 and PSAP, were validated in PDR patients by qPCR analysis. These genes were enriched in pathways such as oxidative phosphorylation and ribosomes, confirming the role of NETs in DR. Upon successful validation, these NETs-associated biomarkers could serve as valuable diagnostic tools and provide guidance for the development of novel therapeutic strategies.

In this study, GBP2 was downregulated in PDR and was identified as a potential risk factor. GBP2 is a member of the guanylate-binding protein (GBP) family. Previous studies have reported that GBP2 can regulate the release of a large number of pro-inflammatory factors such as interleukin (IL) 1β and IL-18 (46). The expression of GBP2 was markedly downregulated in the retina of mice with oxygen-induced retinopathy, which was associated with pathological retinal angiogenesis. Overexpression of GBP2 significantly inhibited neovascularization (47). These findings suggest a potential link between GBP2 levels and DR susceptibility. But the roles of GBPs in DR processes are not completely understood. This research opens a new way to explore GBP2 as a biomarker of PDR and implicated mechanism may be related to oxidative phosphorylation.

In our research, P2RY12 was identified as another potential risk factor for PDR. P2RY12 belongs to the family of G-protein-coupled receptors (48). It is used to regulate platelet activation and aggregation. The over-expression of P2RY12 in the diabetes mellitus lead to platelet aggregation and atherosclerosis development (49). Alterations in platelet that favor thrombosis occur early in the diabetic state and contribute to microvascular disease (50). In recent studies, P2RY12 can also regulate retinal microglia in retinal tissue, which initiate inflammation by releasing proinflammatory cytokines, reactive oxygen species (ROS), and reactive nitrogen species (RNS) (51).

PSAP is a lysosomal regulatory protein. It was downregulated and was identified as a potential protective factor in this study. It was previously reported that silencing of PSAP expression suppressed glycolysis as well as oxidative phosphorylation. Inhibition of PSAP may led to a reduction in atherosclerosis development and in plaque inflammation (52). It was also discovered that knockdown of PSAP strongly inhibited death receptor 6-induced apoptosis (53). But the roles of PSAP in PDR processes are rarely reported.

Our verification test also showed the down-regulated expression of CLIC3 and HOXA1 in DR group though without statistical significance. CLIC3 was a risk factors for PDR, while HOXA1 was found to have protective function. CLIC3 is a member of chloride intracellular channel (CLIC) protein family and may be related to tumor invasion (54). Knockdown of CLIC3 in human gastric cancer cells significantly accelerates cell proliferation. Decreased expression of CLIC3 in gastric cancer may result in poor prognosis of the patients (55). In diabetes mellitus, CLIC3 is significantly downregulated with a result of increased clinical complications (56). HOXA1 is a member of the homeobox (HOX) gene family. The misexpression of HOXA1 in differentiated cells could turn it into an oncogene to participate in cancer development (57). HOXA1 promotes apoptosis, inflammation and phosphorylated NF-κB p65 levels (57, 58). The non-significant results of CLIC3 and HOXA1 may be partly due to the small sample size in the present study.

Several reports have demonstrated that hyperglycemia can trigger sterile inflammation and activate neutrophils (59). The release of NETs by neutrophils can cause endothelial cell damage and vascular remodeling (13). NETs formation can be triggered by various stimuli, including microorganisms, urate crystals, autoantibodies, lipopolysaccharides, ROS, nitric oxide, proinflammatory cytokines, as well as interactions between neutrophils and activated platelets or endothelial cells (9). These findings suggest a reciprocal interaction between sterile inflammation, NETs formation and endothelial dysfunction ultimately leading to a vicious cycle.

There have been reports that the expression of GBP2 and P2RY12 is correlated with neutrophil infiltration (60, 61). The mechanisms that trigger neutrophil infiltration are poorly understood but may result from the release of pro-inflammatory cytokines, such as tumor necrosis factor-α (TNF-α), IL-1β, IL-6, and IL-8 (9). According to previous studies, both GBP2 and P2RY12 can regulate the release of these pro-inflammatory factors (46, 51). PSAP was found to regulate mitogen-activated protein kinase (MAPK), phosphatidylinositol 3’-kinase/AKT serine/threonine kinase 1 (PI3K/Akt) and transforming growth factor-β (TGF-β) pathways (62). These pathways are strongly associated with NETs (63–65). Our research found these biomarkers were enriched in oxidative phosphorylation and ribosomes, suggesting that the upregulation of P2RY2 and the downregulation of GBP2 and PSAP may also regulate NETs by promoting oxidative stress and inflammatory reaction in PDR. All these findings indicate that the NETs related biomarkers play an important role in the pathogenesis of DR.

Furthermore, we predicted drugs targeting P2RY12, among which prasugrel (42, 43), ticagrelor (44) and ticlopidine (42, 45) showed enhanced binding affinity for this biomarker. These newer antiplatelet drugs inhibit platelet aggregation by blocking the P2Y12 platelet receptor. Recent studies have shown that ticagrelor significantly decreases the levels of proinflammatory cytokines, such as TNF-α and IL-6, while increasing the number of circulating endothelial progenitor cells, thereby improving vascular endothelial function (66). Ticlopidine has also been found to increase nitric oxide production in human neutrophils (67). As a result, we predicted that ticagrelor and ticlopidine can be used as a targeted drugs for the treatment of DR. Further studies are needed to elucidate the specific regulatory mechanisms underlying the relationships among P2RY12, the targeted drugs and DR.

There are limitations of the study: Using blood-derived genes may not completely reflect changes in DR-specific tissues like the retina. And the insufficient amount of data and the lack of survival analysis on adequate clinical samples may lead to results bias. Especially, this may be the reason that the expression of HOXA1 and CLIC3 did not reach statistical significance. In the future, we will reach out to more patients and medical institutions to expand the sample size, focusing on an in-depth exploration of specific gene regulation mechanisms in eye tissues to provide a more detailed analysis of their complex regulatory pathways, thereby improving the representativeness and comprehensiveness of the research data. Simultaneously, we are actively exploring collaboration opportunities with external experts and research institutions to gain additional technical support and data analysis resources to further enhance our research capacity.

This study was conducted based on transcriptome self sequencing method, three biomarkers (GBP2, P2RY12 and PSAP) associated with NETs were screened for strong diagnostic value in PDR. In addition, MR analysis revealed that GBP2 and P2RY12 are risk factors for DR and PSAP a protective factor, which may provide a theoretical reference for future studies. However, future longitudinal studies are required to establish whether NETs are formed prior to clinically detectable DR and to determine the diagnostic significance of NETs-related biomarkers for the early detection and stratification of DR patients.

## Data Availability

The data presented in the study are deposited in the NCBI SRA repository, accession number PRJNA1171113.
