# Supplementary material for: Transcriptome sequencing and Mendelian randomization analysis identified biomarkers related to neutrophil extracellular traps in diabetic retinopathy
Source: Front Immunol. 2024 Oct 17;15:1408974. doi: 10.3389/fimmu.2024.1408974 (PMC11524841; doi:10.3389/fimmu.2024.1408974)
Supplement: Supplementary file 1 [file DataSheet1.docx]

| **sample** | **group** | **Age ,y** | **Male/Female** | **Duration of DM ,y** | **HbAc1**  **%** | **FPG**  **mmol/L** | **Number of antidiabetic drugs** |
| --- | --- | --- | --- | --- | --- | --- | --- |
| PDR1 | DR | 46 | M | 16 | 13.0 | 6.67 | 4 |
| PDR2 | DR | 51 | M | 13 | 8.1 | 8.67 | 3 |
| PDR3 | DR | 57 | M | 24 | 10.7 | 8.05 | 4 |
| PDR4 | DR | 49 | M | 15 | 6.7 | 6.29 | 5 |
| PDR5 | DR | 44 | M | 16 | 12.0 | 15.93 | 4 |
| PDR6 | DR | 76 | F | 22 | 10.5 | 10.06 | 4 |
| PDR7 | DR | 70 | F | 17 | 7.5 | 7.44 | 4 |
| PDR8 | DR | 62 | F | 15 | 13.0 | 17.07 | 4 |
| PDR10 | DR | 71 | M | 18 | 11.3 | 7.15 | 4 |
| PDR11 | DR | 66 | M | 18 | 8.7 | 8.70 | 5 |
| N1 | NC | 69 | M | - | - | 4.56 | - |
| N2 | NC | 67 | M | - | - | 5.5 | - |
| N3 | NC | 68 | F | - | - | 4.98 | - |
| N4 | NC | 66 | F | - | - | 5.25 | - |
| N5 | NC | 71 | F | - | - | 4.61 | - |
| N6 | NC | 52 | M | - | - | 4.79 | - |
| N7 | NC | 57 | M | - | - | 5.08 | - |
| N8 | NC | 55 | M | - | - | 5.42 | - |
| N9 | NC | 59 | F | - | - | 5.02 | - |
| N10 | NC | 51 | M | - | - | 5.30 | - |

**Table S1.** The specific sample information.

|  |  |  |  |  |  |  |  |  |  |  |  |  |  |  |
| --- | --- | --- | --- | --- | --- | --- | --- | --- | --- | --- | --- | --- | --- | --- |
|  |  | **SNP** | **effect_allele.**  **exposure** | **other_allele.**  **exposure** | **beta.exposure** | **se.exposure** | **pval.exposure** | **beta.outcome** | **se.outcome** | **pval.outcome** | **eaf.exposure** | **F** | **R2** | **exposure** |
|  | s10275896 | rs10275896 | C | T | 0.162849 | 0.014224 | 2.37E-30 | -0.04731 | 0.018376 | 0.010033 | 0.223386 | 131.0863 | NA | eqtl-a-ENSG00000105991 |
|  | rs1082315 | rs1082315 | G | C | 0.111027 | 0.017817 | 4.62E-10 | 0.003518 | 0.022337 | 0.874866 | 0.12755 | 38.83141 | NA | eqtl-a-ENSG00000105991 |
|  | rs2106904 | rs2106904 | T | C | -0.07274 | 0.013293 | 4.44E-08 | 0.02726 | 0.017209 | 0.113185 | 0.276463 | 29.94494 | NA | eqtl-a-ENSG00000105991 |
|  | rs774267 | rs774267 | C | T | 0.265505 | 0.011697 | 4.56E-114 | -0.03947 | 0.014462 | 0.006353 | 0.513929 | 515.2685 | NA | eqtl-a-ENSG00000105991 |
|  | rs10876550 | rs10876550 | A | G | -0.11631 | 0.012062 | 5.28E-22 | 0.00214 | 0.015483 | 0.890094 | 0.590064 | 92.98275 | NA | eqtl-a-ENSG00000169313 |
|  | rs114694170 | rs114694170 | C | T | -0.19953 | 0.026677 | 7.45E-14 | -0.03726 | 0.03108 | 0.230549 | 0.052318 | 55.94145 | NA | eqtl-a-ENSG00000169313 |
|  | rs1434282 | rs1434282 | T | C | 0.078252 | 0.013118 | 2.44E-09 | 0.022574 | 0.015866 | 0.154808 | 0.711362 | 35.58271 | NA | eqtl-a-ENSG00000169313 |
|  | rs2172249 | rs2172249 | C | G | -0.28865 | 0.012302 | 9.75E-122 | -0.03103 | 0.015748 | 0.048754 | 0.657254 | 550.5142 | NA | eqtl-a-ENSG00000169313 |
|  | rs342296 | rs342296 | A | G | 0.094145 | 0.011961 | 3.52E-15 | -0.01705 | 0.01433 | 0.234203 | 0.441001 | 61.95286 | NA | eqtl-a-ENSG00000169313 |
|  | rs6136489 | rs6136489 | G | T | -0.07245 | 0.012511 | 6.99E-09 | 0.000792 | 0.01588 | 0.960212 | 0.344366 | 33.53677 | NA | eqtl-a-ENSG00000169313 |
|  | rs6993770 | rs6993770 | T | A | -0.09306 | 0.013323 | 2.85E-12 | -0.02055 | 0.017276 | 0.23427 | 0.273922 | 48.79048 | NA | eqtl-a-ENSG00000169313 |
|  | rs7612010 | rs7612010 | G | A | -0.19581 | 0.011899 | 7.70E-61 | -0.01203 | 0.014508 | 0.40685 | 0.567289 | 270.7799 | NA | eqtl-a-ENSG00000169313 |
|  | rs8073060 | rs8073060 | A | T | -0.10675 | 0.012887 | 1.20E-16 | -0.01703 | 0.016678 | 0.307242 | 0.305723 | 68.61258 | NA | eqtl-a-ENSG00000169313 |
|  | rs11570034 | rs11570034 | G | A | -1.09005 | 0.031191 | 1.00E-200 | -0.0777 | 0.037503 | 0.03828 | 0.034717 | 1221.341 | NA | eqtl-a-ENSG00000066336 |
|  | rs11570071 | rs11570071 | A | C | -0.74027 | 0.036764 | 3.59E-90 | -0.12718 | 0.04564 | 0.005328 | 0.026161 | 405.441 | NA | eqtl-a-ENSG00000066336 |
|  | rs117082751 | rs117082751 | A | G | 0.245168 | 0.037939 | 1.03E-10 | 0.072909 | 0.056863 | 0.199778 | 0.025172 | 41.75999 | NA | eqtl-a-ENSG00000066336 |
|  | rs1471934 | rs1471934 | T | C | 0.202598 | 0.034149 | 2.98E-09 | -0.06678 | 0.049067 | 0.173523 | 0.031279 | 35.19812 | NA | eqtl-a-ENSG00000066336 |
|  | rs7114704 | rs7114704 | T | C | 0.243729 | 0.026462 | 3.24E-20 | 0.073533 | 0.024139 | 0.002317 | 0.053108 | 84.83704 | NA | eqtl-a-ENSG00000066336 |
|  | rs149007767 | rs149007767 | T | C | 0.126555 | 0.016648 | 2.92E-14 | 0.00602 | 0.017848 | 0.735885 | 0.149708 | 57.79035 | NA | eqtl-a-ENSG00000162645 |
|  | rs2182322 | rs2182322 | G | A | 0.233605 | 0.033722 | 4.29E-12 | 0.025891 | 0.032276 | 0.422449 | 0.967926 | 47.98883 | NA | eqtl-a-ENSG00000162645 |
|  | rs4788084 | rs4788084 | T | C | -0.08627 | 0.012038 | 7.72E-13 | -0.00882 | 0.014373 | 0.539562 | 0.419635 | 51.35028 | NA | eqtl-a-ENSG00000162645 |
|  | rs653178 | rs653178 | T | C | -0.17708 | 0.011848 | 1.66E-50 | -0.10099 | 0.014462 | 2.88E-12 | 0.539991 | 223.3698 | NA | eqtl-a-ENSG00000162645 |
|  | rs76830965 | rs76830965 | A | C | 0.120281 | 0.018856 | 1.78E-10 | -0.01799 | 0.025842 | 0.486355 | 0.111855 | 40.69068 | NA | eqtl-a-ENSG00000162645 |
|  | rs115292410 | rs115292410 | C | T | -0.24966 | 0.02369 | 5.75E-26 | 0.011325 | 0.033597 | 0.736049 | 0.067132 | 111.0563 | NA | eqtl-a-ENSG00000163393 |
|  | rs149110519 | rs149110519 | T | C | 0.161939 | 0.026623 | 1.18E-09 | -0.04907 | 0.045135 | 0.276911 | 0.052616 | 36.99919 | NA | eqtl-a-ENSG00000163393 |
|  | rs2811577 | rs2811577 | T | C | 0.276469 | 0.015937 | 2.06E-67 | -0.04852 | 0.018483 | 0.008667 | 0.163182 | 300.9323 | NA | eqtl-a-ENSG00000163393 |
|  | rs10980797 | rs10980797 | G | A | -0.07233 | 0.011906 | 1.24E-09 | 0.00733 | 0.014331 | 0.609018 | 0.472236 | 36.90953 | NA | eqtl-a-ENSG00000197746 |
|  | rs1491105191 | rs149110519 | T | C | 0.150688 | 0.026628 | 1.52E-08 | -0.04907 | 0.045135 | 0.276911 | 0.052616 | 32.02531 | NA | eqtl-a-ENSG00000197746 |
|  | rs4747202 | rs4747202 | A | G | 0.333322 | 0.020604 | 7.23E-59 | -0.06425 | 0.022404 | 0.004134 | 0.090033 | 261.7254 | NA | eqtl-a-ENSG00000197746 |
|  | rs112733823 | rs112733823 | T | C | 0.168646 | 0.019921 | 2.55E-17 | 0.104327 | 0.017075 | 9.97E-10 | 0.098516 | 71.66803 | NA | eqtl-a-ENSG00000206503 |
|  | rs1532624 | rs1532624 | A | C | -0.07601 | 0.011982 | 2.24E-10 | -0.00798 | 0.014585 | 0.584459 | 0.437102 | 40.24284 | NA | eqtl-a-ENSG00000206503 |
|  | rs1611638 | rs1611638 | C | G | -0.74137 | 0.024161 | 1.00E-200 | -0.03123 | 0.035034 | 0.372721 | 0.060554 | 941.5484 | NA | eqtl-a-ENSG00000206503 |
|  | rs2844841 | rs2844841 | C | A | 0.461157 | 0.012192 | 1.00E-200 | 0.117823 | 0.014661 | 9.25E-16 | 0.316665 | 1430.791 | NA | eqtl-a-ENSG00000206503 |
|  | rs72863525 | rs72863525 | A | C | -0.13604 | 0.020915 | 7.79E-11 | -0.1117 | 0.033741 | 0.000931 | 0.088582 | 42.30857 | NA | eqtl-a-ENSG00000206503 |
|  | rs9258357 | rs9258357 | C | T | 0.268941 | 0.012695 | 1.34E-99 | 0.069383 | 0.016227 | 1.91E-05 | 0.692319 | 448.7744 | NA | eqtl-a-ENSG00000206503 |
|  | rs9391683 | rs9391683 | A | G | -0.09688 | 0.012518 | 1.00E-14 | -0.12765 | 0.014937 | 1.28E-17 | 0.342272 | 59.89492 | NA | eqtl-a-ENSG00000206503 |
|  | rs1491105192 | rs149110519 | T | C | -0.18715 | 0.026611 | 2.02E-12 | -0.04907 | 0.045135 | 0.276911 | 0.052616 | 49.46173 | NA | eqtl-a-ENSG00000105339 |
|  | rs186287 | rs186287 | C | T | -0.18784 | 0.012336 | 2.33E-52 | 0.018962 | 0.015312 | 0.21557 | 0.644826 | 231.8721 | NA | eqtl-a-ENSG00000105339 |
|  | rs28444357 | rs28444357 | G | A | -0.08664 | 0.011881 | 3.04E-13 | 0.009357 | 0.014471 | 0.517879 | 0.498537 | 53.18184 | NA | eqtl-a-ENSG00000105339 |
|  | rs56330463 | rs56330463 | C | T | 0.077639 | 0.01205 | 1.17E-10 | -0.01474 | 0.014854 | 0.321106 | 0.582097 | 41.51591 | NA | eqtl-a-ENSG00000105339 |
|  | rs9792 | rs9792 | G | A | -0.41451 | 0.011562 | 1.00E-200 | 0.024521 | 0.014505 | 0.090935 | 0.415607 | 1285.349 | NA | eqtl-a-ENSG00000105339 |
|  | rs111378975 | rs111378975 | T | C | 0.21357 | 0.028786 | 1.18E-13 | -0.04971 | 0.037878 | 0.189441 | 0.044569 | 55.04461 | NA | eqtl-a-ENSG00000135114 |
|  | rs1169286 | rs1169286 | C | T | 0.462754 | 0.011378 | 1.00E-200 | 0.043891 | 0.01461 | 0.002662 | 0.428877 | 1654.271 | NA | eqtl-a-ENSG00000135114 |
|  | rs1490077671 | rs149007767 | T | C | 0.164792 | 0.016624 | 3.66E-23 | 0.00602 | 0.017848 | 0.735885 | 0.149708 | 98.26789 | NA | eqtl-a-ENSG00000135114 |
|  | rs2071190 | rs2071190 | A | T | 0.283053 | 0.013659 | 2.18E-95 | -0.0097 | 0.017481 | 0.579127 | 0.243592 | 429.4164 | NA | eqtl-a-ENSG00000135114 |
|  | rs2749592 | rs2749592 | A | C | 0.089012 | 0.012831 | 3.99E-12 | -0.02607 | 0.014707 | 0.076324 | 0.311446 | 48.12924 | NA | eqtl-a-ENSG00000135114 |
|  | rs28498283 | rs28498283 | T | A | 0.078659 | 0.013651 | 8.31E-09 | 0.01703 | 0.01636 | 0.2979 | 0.254318 | 33.20156 | NA | eqtl-a-ENSG00000135114 |
|  | rs4917014 | rs4917014 | G | T | -0.08633 | 0.012776 | 1.41E-11 | 0.009776 | 0.015579 | 0.530315 | 0.316454 | 45.65551 | NA | eqtl-a-ENSG00000135114 |
|  | rs509150 | rs509150 | T | G | -0.21685 | 0.026408 | 2.19E-16 | -0.00319 | 0.03169 | 0.919871 | 0.946596 | 67.42691 | NA | eqtl-a-ENSG00000135114 |
|  | rs57994353 | rs57994353 | C | T | -0.07464 | 0.013038 | 1.04E-08 | -0.03691 | 0.01642 | 0.024574 | 0.294804 | 32.77197 | NA | eqtl-a-ENSG00000135114 |
|  | rs11920354 | rs11920354 | A | C | -0.06727 | 0.012295 | 4.46E-08 | -0.02906 | 0.014901 | 0.051192 | 0.372903 | 29.93531 | NA | eqtl-a-ENSG00000169583 |
|  | rs2256183 | rs2256183 | G | A | 0.070477 | 0.012033 | 4.71E-09 | -0.00963 | 0.014486 | 0.506388 | 0.577131 | 34.30322 | NA | eqtl-a-ENSG00000169583 |
|  | rs2617170 | rs2617170 | C | T | -0.08687 | 0.012617 | 5.80E-12 | -0.01415 | 0.01516 | 0.350538 | 0.668027 | 47.39808 | NA | eqtl-a-ENSG00000169583 |
|  | rs7911264 | rs7911264 | C | T | -0.1086 | 0.011874 | 5.92E-20 | -0.06243 | 0.014339 | 1.34E-05 | 0.515223 | 83.64517 | NA | eqtl-a-ENSG00000169583 |
|  | rs908839 | rs908839 | G | C | -0.33325 | 0.011569 | 1.85E-182 | 0.009096 | 0.014628 | 0.534066 | 0.505534 | 829.7328 | NA | eqtl-a-ENSG00000169583 |
|  | rs146357591 | rs146357591 | T | C | -0.35246 | 0.03918 | 2.34E-19 | -0.0053 | 0.041032 | 0.897161 | 0.023497 | 80.92995 | NA | eqtl-a-ENSG00000204642 |
|  | rs15326241 | rs1532624 | A | C | -0.11606 | 0.011959 | 2.86E-22 | -0.00798 | 0.014585 | 0.584459 | 0.437102 | 94.19012 | NA | eqtl-a-ENSG00000204642 |
|  | rs16894836 | rs16894836 | G | T | 0.12945 | 0.017166 | 4.67E-14 | 0.130263 | 0.020029 | 7.83E-11 | 0.139063 | 56.86508 | NA | eqtl-a-ENSG00000204642 |
|  | rs3130248 | rs3130248 | T | C | -0.43647 | 0.011325 | 1.00E-200 | 0.153899 | 0.01434 | 7.21E-27 | 0.490434 | 1485.407 | NA | eqtl-a-ENSG00000204642 |
|  | rs3132681 | rs3132681 | A | C | 0.338834 | 0.01197 | 2.92E-176 | 0.019638 | 0.014428 | 0.173484 | 0.373258 | 801.2423 | NA | eqtl-a-ENSG00000204642 |
|  | rs9258116 | rs9258116 | C | T | -0.20742 | 0.013012 | 3.34E-57 | -0.10972 | 0.018301 | 2.03E-09 | 0.289065 | 254.0771 | NA | eqtl-a-ENSG00000204642 |
|  | rs92583571 | rs9258357 | C | T | -0.18938 | 0.012797 | 1.49E-49 | 0.069383 | 0.016227 | 1.91E-05 | 0.692319 | 219.0072 | NA | eqtl-a-ENSG00000204642 |
|  | rs9468618 | rs9468618 | T | C | 0.738026 | 0.020808 | 1.00E-200 | -0.19468 | 0.032507 | 2.11E-09 | 0.081812 | 1257.994 | NA | eqtl-a-ENSG00000204642 |

**Table S2.** The F statistics values for these 11 candidate biomarkers

| Gene_1 | Gene_2 | cor | p.value |
| --- | --- | --- | --- |
| HOXA1 | GBP2 | 0.280206 | 0.231467 |
| HOXA1 | P2RY12 | -0.26609 | 0.256803 |
| HOXA1 | CLIC3 | 0.330718 | 0.154381 |
| HOXA1 | PSAP | 0.572329 | 0.008363 |
| GBP2 | P2RY12 | -0.64086 | 0.00233 |
| GBP2 | CLIC3 | 0.483328 | 0.030857 |
| GBP2 | PSAP | 0.660282 | 0.001533 |
| P2RY12 | CLIC3 | -0.52957 | 0.016335 |
| P2RY12 | PSAP | -0.36065 | 0.118261 |
| CLIC3 | PSAP | 0.406777 | 0.075089 |

**Table S3.** The correlation analysis between the genes
