# Supplementary figures and images for: Transcriptome sequencing and Mendelian randomization analysis identified biomarkers related to neutrophil extracellular traps in diabetic retinopathy
Source: Front Immunol. 2024 Oct 17;15:1408974. doi: 10.3389/fimmu.2024.1408974 (PMC11524841; doi:10.3389/fimmu.2024.1408974)

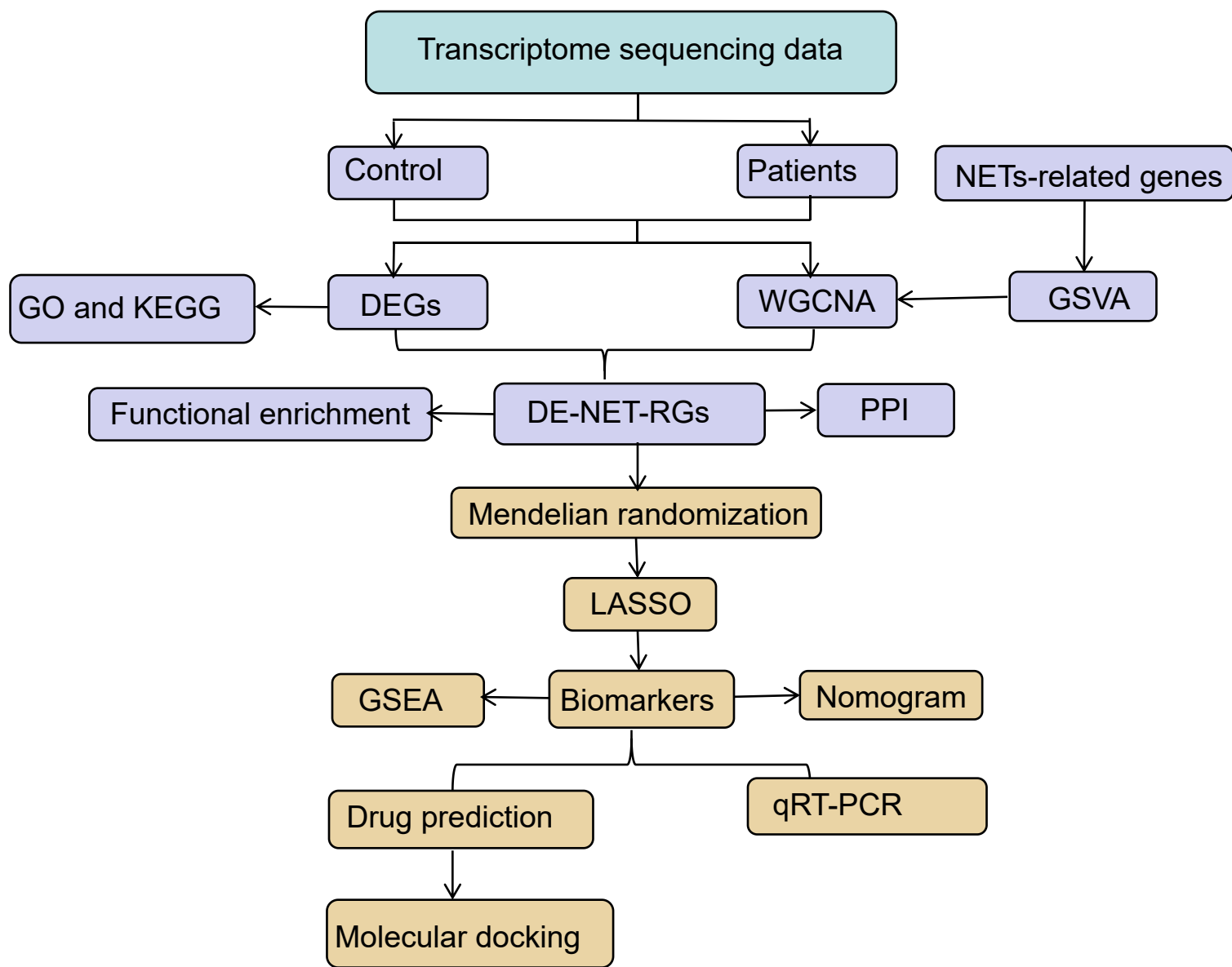

Supplement: Supplementary file 2 [file DataSheet2.pdf]
